# Molecular Diagnostics for Gonorrhoea: Implications for Antimicrobial Resistance and the Threat of Untreatable Gonorrhoea

**DOI:** 10.1371/journal.pmed.1001598

**Published:** 2014-02-04

**Authors:** Nicola Low, Magnus Unemo, Jørgen Skov Jensen, Judith Breuer, Judith M. Stephenson

**Affiliations:** 1 Institute of Social and Preventive Medicine, University of Bern, Bern, Switzerland; 2 World Health Organization Collaborating Centre for Gonorrhoea and other Sexually Transmitted Infections, Örebro University Hospital, Örebro, Sweden; 3 Department of Microbiology and Infection Control, Statens Serum Institut, Copenhagen, Denmark; 4 MRC-UCL Centre for Medical Molecular Virology, Division of Infection and Immunity, University College London, London, United Kingdom; 5 Institute for Women's Health, University College London, London, United Kingdom

## Abstract

This Essay from Nicola Low and colleagues discusses the importance of the nucleic acid amplification tests for rapid detection of N. gonorrhoeae and its resistance determinants, as well as the importance of ensuring their rational use, as priorities for controlling both gonorrhoea and antimicrobial resistance.

*Please see later in the article for the Editors' Summary*

**Summary Points**

- Antimicrobial resistance and overuse of antimicrobials are serious threats to the treatment of gonorrhoea.
- *Neisseria gonorrhoeae* susceptibility to extended spectrum cephalosporins is decreasing and treatment failures are spreading, but no new drug class is licensed to replace them for immediate treatment.
- Nucleic acid amplification tests are increasingly used to diagnose gonorrhoea but current commercially available tests do not detect antimicrobial resistance.
- Tests for gonorrhoea that allow individually tailored antimicrobial therapy at the first contact with health services will need to be point-of-care tests that can be integrated into the diagnostic process to give accurate results in around an hour.
- Development of nucleic acid amplification tests that incorporate rapid detection of *N. gonorrhoeae* and its resistance determinants and ensuring the rational use of antimicrobials are priorities for controlling both gonorrhoea and antimicrobial resistance.

Antimicrobial resistance (AMR) is making the clinical management of infections such as gonorrhoea increasingly difficult worldwide [1]–[3]. In between the discovery of penicillin and the emergence of multidrug resistant (MDR-NG) and extensively drug resistant (XDR-NG) strains [4],[5], gonorrhoea was considered unpleasant, but not particularly serious, because it was easily treated [4]–[7]. Experts increasingly describe *N. gonorrhoeae* as becoming an untreatable superbug [2],[4],[7] because of reports of MDR-NG and XDR-NG strains resulting in treatment failures with extended spectrum cephalosporins (ESCs), such as cefixime and ceftriaxone, from Europe, North America, Asia, and Africa [7]–[9]. The World Health Organization (WHO) estimated that there were 106 million new uncomplicated gonococcal infections worldwide in 2008, the majority of cases in places with limited diagnostic and treatment options. If these infections become untreatable then complications, including pelvic inflammatory disease, ectopic pregnancy, tubal infertility, neonatal eye infections, and consequences such as facilitation of HIV co-transmission, will become more common [2],[5].

The emergence of ESC resistance has coincided with the rapid expansion of molecular diagnostic testing for gonorrhoea (Figure 1). PCR is an invention that revolutionised diagnostic testing in infectious diseases, as in many other fields of medicine. Diagnostic tests that amplify specific DNA or RNA sequences are known collectively as nucleic acid amplification tests (NAATs) and include PCR. The benefits of NAATs for pathogen detection are widely recognised [1]. There are now commercially available NAATs for rapid detection of tuberculosis, including rifampicin resistance [10], and for methicillin resistant *Staphylococcus aureus* (MRSA) and its main resistance gene [11]. Experience with rapid molecular diagnostics shows that the potential health gains are determined not by the technology but by its successful implementation in a diagnostic process [11],[12]. For tuberculosis, the time to diagnosis could be shortened from weeks to hours if the rapid test can be placed at the point of care. At present, culture-based antimicrobial susceptibility testing is still needed because the specificity of the rapid NAATs is too low to allow definitive decisions about treatment of either tuberculosis [10] or MRSA [11]. Mathematical modelling suggests that the impact of rapid molecular diagnosis of tuberculosis in high HIV prevalence areas might be less than anticipated because the major gain is in early detection of less infectious smear-negative tuberculosis and the large reservoir of latent infection remains undetected [13].

**Figure 1:**
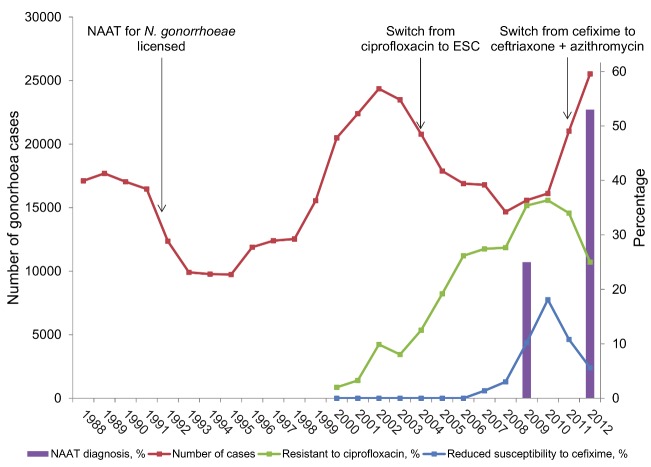
Trend in number of cases of gonorrhoea diagnosed, NAAT testing for gonorrhoea, and selected antimicrobial resistance in genitourinary medicine clinics in England and Wales. Cases of gonorrhoea are from Public Health England; percentages of tests done using NAATs are from UK audits of asymptomatic patients in genitourinary medicine clinics (http://www.bashh.org/BASHH/BASHH_Groups/National_Audit_Group/BASHH/BASHH_Groups/National_Audit_Group.aspx?hkey=c17918b8-5c72-40bd-981f-632f89e45708). Percentage of isolates resistant to ciprofloxacin and cefixime are from reference [1] and the 2012 report of the UK Gonococcal Resistance to Antimicrobials Surveillance Programme (GRASP, http://www.hpa.org.uk/Topics/InfectiousDiseases/InfectionsAZ/Gonorrhoea/AntimicrobialResistance/), with values obtained using PlotDigitizer software (http://plotdigitizer.sourceforge.net/).

The limitations of molecular diagnostic tests for sexually transmitted infections are less widely publicised and tend to focus on diagnostic test performance issues [14]–[17]. In this Essay we discuss the benefits and disadvantages of molecular diagnosis of *N. gonorrhoeae* in the context of AMR and the implications of AMR for gonorrhoea treatment recommendations.

## Current Management of Gonorrhoea

Prompt and appropriate treatment for patients with gonorrhoea and their sexual partners at the first contact with health services is the priority, endorsed by international guidelines [18]. Delaying treatment to wait for diagnostic and antimicrobial susceptibility results is unacceptable for three reasons. First, urethritis, the most common clinical presentation in men, can cause purulent urethral discharge and severe pain, typically described as “pissing glass” [19]. Second, sexual partners of people with diagnosed gonorrhoea have a very high risk of being infected [20]; rapid treatment is especially important for preventing re-infection and reducing onward transmission in groups with the highest rates of sexual partner change and AMR gonorrhoea, such as men who have sex with men and sex workers. Third, most gonococcal infections are diagnosed in settings with no, or limited, diagnostic facilities. WHO syndromic management flow charts recommend empirical antimicrobial treatment that should cure 95% or more cases of the most common causes of the syndrome [18]. The syndromic approach is reasonably accurate for the management of gonococcal urethritis in men but does not work well for cervical gonorrhoea in women or rectal gonorrhoea in men who have sex with men and women because most of these infections are asymptomatic [21]. Immediate treatment for symptomatic infections is a useful strategy when antimicrobials can be replaced once the cure rate falls below the threshold. But overuse of antimicrobials is now a serious problem for gonorrhoea because ESCs are “last-line” antimicrobials and no new drug classes are licensed to replace them [2],[7].

Where laboratory facilities are available, culture is a sensitive method for the diagnosis of genital *N. gonorrhoeae* infections and is completely specific if adequate confirmatory tests are done [20]. *N. gonorrhoeae* has fastidious growth requirements so highly nutritious selective culture media are needed (Table 1). Antimicrobial susceptibility can then be tested on cultured isolates but is, unfortunately, not always done. The laboratory tests for culture and susceptibility take 48 hours in total. Strengthening culture-based surveillance of local AMR patterns is an increasingly important tool for the detection of resistant strains and monitoring their spread [2], even if routine culture facilities are not available.

**Table 1:** Advantages and disadvantages of molecular diagnostic testing for gonorrhoea in relation to culture and antimicrobial susceptibility testing.

Characteristic	Advantage of NAATs	Disadvantage of NAATs
Ease of testing	Can be done on non-invasive and self-collected specimens.	More testing in low risk populations; low positive predictive value results in unnecessary treatment and potential harm to personal relationships.
Case detection	More cases diagnosed. More accessible testing for hard to reach groups at high risk of infection, e.g., sex workers, men who have sex with men.	Gains in increased case detection over culture could be offset if treating more cases results in more people who are susceptible to re-infection.
Test performance	More sensitive than culture, especially in pharyngeal, rectal, and asymptomatic infections.	Specificity decreased by cross-reactions and other sequence-related issues.
Laboratory requirements	Automation allows high throughput, reduces contamination.	Expensive equipment and specialised training required.
AMR	In-house assays detect some AMR mutations and resistant strains.	No current antimicrobial susceptibility testing in commercial tests; Complete AMR testing cannot be performed.
Treatment failure		Detection relies on clinical treatment failure or a late test of cure.
Licensing approval	Approved for endocervical, vaginal, urethral, urine specimens	Not approved for pharyngeal or rectal specimens but can be used if laboratory evaluation satisfactory.

## Advantages of Molecular Diagnostics for Gonorrhoea

There is no commercially available test for gonorrhoea that gives both same-day diagnosis and an antimicrobial susceptibility profile for *N. gonorrhoeae*. But the ability of NAATs to detect tiny amounts of nucleic acid has several advantages over culture for the diagnosis of an *N. gonorrhoeae* infection. First, specimens for NAATs are easier to transport and store because they do not need the organism to be viable for detection. Second, NAATs can be automated and multiplexed detecting both *Chlamydia trachomatis* and *N. gonorrhoeae*, which is useful because both organisms cause similar clinical syndromes. Third, analysis can be done on non-invasive self-collected specimens like urine and vaginal swabs. These qualities mean that testing is easier in remote areas and can be extended to groups who were previously hard to reach but at high risk of both infection and AMR, such as men who have sex with men [3]. Fourth, NAATs are more sensitive than culture methods in general and particularly for asymptomatic infections in the pharynx and rectum (although no internationally available commercial NAAT has licensing approval for use on extra-genital samples) [22].

NAATs for *N. gonorrhoeae* diagnosis became available in the early 1990s [22] and are now the most common method used for gonorrhoea diagnosis in many countries, including the UK (Figure 1) and US [23]. The sharp increase in the number of diagnosed gonorrhoea cases since 2010 (Figure 1) is likely to be associated, in part, with both higher numbers of tests and the higher sensitivity of NAAT. In the US, the absolute number of gonorrhoea tests, estimated from data from manufacturers, increased from 2000 to 2004 but the percentage of tests that were done by culture fell [23].

## Disadvantages of Molecular Diagnostics for Gonorrhoea

### NAATs do not routinely provide information about antimicrobial susceptibility of N. gonorrhoeae

Many of the limitations of NAATs for gonorrhoea diagnosis are the flip-side of their advantages. The main limitation for clinical management is that there is no viable organism so NAATs cannot provide data about minimum inhibitory concentrations for antimicrobials, which guide therapy [2],[3],[7],[14],[24]. If the health care professional takes a culture specimen and requests antimicrobial susceptibility testing that shows that the organism is resistant to the antibiotic prescribed, they can recall the patient within 2 to 3 days and prescribe individually tailored antibiotics. Or, if the culture-based test on an asymptomatic patient is positive, antimicrobial susceptibility testing allows specific antimicrobial therapy to be prescribed. All results from NAAT-diagnosed gonococcal infections have to be treated “blind” and commercially available NAATs do not currently detect any AMR determinants, so more NAAT-diagnosed than culture-diagnosed infections will be unnecessarily treated with ESC-based regimens.

Molecular methods for AMR *N. gonorrhoeae* could give results faster (2–3 hours) than culture-based testing (48 hours). But the actual turnaround time, from ordering the test to changing patient care, is not much faster if the molecular test is run on batched samples once a day or every other day [11]. Tests for gonorrhoea that allow individually tailored antimicrobial therapy at the first contact with health services will need to be point-of-care tests with an actual turnaround time of around an hour [25]. The weak correlation between *N. gonorrhoeae* genetic resistance determinants, minimum inhibitory concentrations of ESC, and treatment outcome currently makes it difficult to use genetic markers to guide therapy for gonorrhoea [7].

The first documented case of XDR-NG was diagnosed in a Japanese female sex worker who had a pharyngeal infection diagnosed initially by NAAT. A culture specimen was obtained two weeks later when she returned for treatment but there was no post-treatment culture specimen and she was lost to follow-up for three months, after receiving a second dose of ceftriaxone for presumed treatment failure diagnosed by NAAT [4],[26]. This case report shows how detection of AMR and treatment failure in NAAT-diagnosed gonorrhoea infections can be delayed. AMR will only be detected if the patient returns for a follow-up visit and a culture specimen is taken or if the patient returns with persistent symptoms. But antimicrobial resistant infections can persist asymptomatically. Pharyngeal gonorrhoea is often asymptomatic, might require higher doses of antibiotics to cure it [5], and is the location that is most difficult to diagnose by culture because of growth of other bacterial species and low bacterial load [27]. Many patients with gonorrhoea do not attend for a follow-up visit at all [28]. Resistant strains of gonorrhoea can therefore spread undetected if patients have not had a culture specimen taken at initial presentation and do not attend for follow-up.

### NAATs encourage over-testing and overtreatment of gonorrhoea

Simplified specimen collection and multiplex testing have disadvantages. Over-testing is facilitated by simultaneous testing for *N. gonorrhoeae* on specimens taken for chlamydia screening in populations at low risk of gonorrhoea such as asymptomatic heterosexual adults tested in primary care [15]. In such settings, the predictive value of a positive test for gonorrhoea can be unacceptably low [14],[17], meaning that most people with a positive test are not infected. Overtreatment will occur if clinicians interpret initial positive gonorrhoea NAAT results as diagnosed infections without supporting information from a sexual history and/or a confirmatory test, as recommended [14],[20]. The negative consequences of false-positive diagnosis of gonorrhoea in a low risk population include breakdown of previously stable monogamous relationships [14]. Whilst some NAATs for *N. gonorrhoeae* show very high specificity [29], their performance is inherently limited by genetic sequence variation between subtypes and cross-reactions with related *Neisseria* species [22]. Overtreatment also has consequences for AMR. Unnecessary use of extended spectrum antimicrobials, such as ceftriaxone, increases the chances that commensal *Neisseria spp.* develop resistance and that resistance determinants will be transferred horizontally to *N. gonorrhoeae* [22],[30].

### The cost of NAAT for gonorrhoea diagnosis

The high cost of NAATs can result in over-testing or under-testing, depending on who has to pay for the test and who makes the profit. Over-testing with NAATs occurs if health care systems reimburse laboratories for the number of analytic targets. Laboratories can charge twice for a NAAT to detect *N. gonorrhoeae* and *C. trachomatis* even if the tests are done simultaneously in the same automated procedure [15]. Under-testing can occur if the patient has to pay an out of pocket contribution, e.g., in primary care in the Netherlands (J van Bergen, 02.07.2013, personal communication) and in the Swiss health system (D. Oertle, 03.07.2013, personal communication). Patients at high risk of sexually transmitted infections might be unwilling to have check-ups if they are asymptomatic. Patients with symptoms might decline laboratory testing and opt for syndromic treatment with antimicrobials to cover the most common causative organisms. In the latter situation, blind antibiotic treatment and loss to follow-up could exacerbate the spread of AMR, which will, however, be undetected.

### Antimicrobial prescribing for gonorrhoea and AMR

Antimicrobial prescribing policies can also contribute to the emergence of AMR gonorrhoea [2],[7],[24]. Single dose oral treatment with a single class of antimicrobials is a goal of selecting drugs for sexually transmitted infections, including gonorrhoea [2] to aid adherence to recommended regimens by both patients and health care professionals [24]. Monotherapy, however, exerts selection pressure for resistance to emerge [24]. The WHO criterion for selecting regimens for which microbial resistance is “unlikely to develop or can be delayed” [2] is probably incompatible with the desire for single dose oral monotherapy. Efficacy of at least 95% is required for a first line antimicrobial that will be used for empiric treatment [2]. Cefixime was the last available single dose oral treatment and AMR surveillance data show that this no longer has the required level of efficacy (Figure 1). Clinical guidelines in Europe [20] and the US [31] now recommend combination treatment with intramuscular ceftriaxone and oral azithromycin. The addition of azithromycin is to delay the emergence of resistance to ESC [20],[24],[31]. High level resistance to azithromycin monotherapy has already emerged in the treatment of gonorrhoea, syphilis, chancroid, and *Mycoplasma genitalium* and experts have called for its use to treat all bacterial sexually transmitted infections to be limited [24].

## The Role of Molecular Diagnosis in the Spread and Control of Drug Resistant Gonorrhoea

Could the expansion of NAAT diagnosis for gonorrhoea exacerbate the continuing spread of AMR gonorrhoea? There is no definitive answer to this question yet but there are issues that should be investigated further, given the global increase in AMR, the lack of new first line antimicrobials for gonorrhoea treatment [1], and the continued expansion of NAAT at the expense of culture-based testing [2],[7],[23]. Countries with strong gonococcal antimicrobial surveillance systems and antimicrobial stewardship policies can mitigate the potentially negative impacts of a diagnostic shift to NAATs [3]. But misuse and abuse of antimicrobials are widespread in many countries, and there is no strong surveillance of AMR, even in Japan where AMR is often detected first [5].

Another reasonable question is whether diagnostic test manufacturers, regulators, and researchers should have considered the implications that NAATs for gonorrhoea diagnosis might have on AMR before promoting and expanding their use so widely. The benefits of NAAT diagnosis for sexually transmitted infections often consider gonorrhoea and chlamydia together. The gains in sensitivity and technical complexity of NAATs compared to culture are smaller for *N. gonorrhoeae* diagnosis than for *C. trachomatis* [14], and AMR in chlamydia remains largely unexplored [24]. Early descriptions of commercial NAAT development for *N. gonorrhoeae* do not appear to mention the inability to give AMR data as a potential problem [32]. This is surprising as gonococcal resistance was already a global public health issue at the time of development.

Academic research groups have developed in-house molecular tests for one or more genetic determinants of AMR *N. gonorrhoeae* [33]–[35]. But very few of these tests detect mutations associated with [36], or strains exhibiting [37], ESC resistance, which is the imminent threat. The ongoing evolution of ESC resistance, involving combinations of mutations in several genes, is a major challenge for test development. Tests that need continual updating with new target sequences are unlikely to be profitable for diagnostics companies in the short term. Supranational initiatives to invest in non-profit research might therefore be needed to overcome some of the challenges of feasibility and commercial viability.

## Conclusions

Current knowledge and practice about the use of NAAT for gonorrhoea diagnosis could be improved in several ways. First, all national and international guidelines about the use of NAAT for gonorrhoea diagnosis should specify situations in which culture-based testing and tests of cure are needed; transport media that can be used for both NAAT and culture will facilitate this. The cost of additional testing should be borne by the health system because the information about AMR would be used for the public health good. Second, in the absence of empirical diagnostic trials, mathematical modelling will be needed to explore the impact of NAAT-based rapid tests for the detection of AMR gonorrhoea on the spread of resistance and on clinical outcomes. Dynamic transmission models can capture the net effects of competing factors such as increased detection and treatment of gonorrhoea, increased re-infection risk, and reduced or delayed detection of AMR on the transmission of gonorrhoea and of resistant strains. Third, clinicians should follow international guidelines for the early detection of ESC resistant gonorrhoea and clinical treatment failure, which take into account the role of NAAT diagnosis [2]. Fourth, the development of molecular tests to detect gonococcal resistance mutations should become part of the solution [1],[2],[7],[25]. Commercial diagnostics companies should invest more to develop and evaluate NAATs that detect both *N. gonorrhoeae* and AMR determinants reliably, particularly in resource poor settings. The true cost of ignoring gonococcal AMR will include the costs of treating the infection and its complications as experienced in the pre-antibiotic era [38]. AMR gonorrhoea needs to be conceptualised and tackled as part of the global problem of resistance [1],[2] with strong proactive programs for phenotypic AMR surveillance to monitor and even pre-empt the emergence of critical levels of AMR [3]. Improving the capabilities of NAAT diagnosis for gonorrhoea and ensuring their rational use is a priority for controlling both gonorrhoea and AMR.
